# Retraction Note: Effectiveness and safety of sorafenib for renal cell, hepatocellular and thyroid carcinoma: pooled analysis in patients with renal impairment

**DOI:** 10.1007/s00280-023-04587-8

**Published:** 2023-09-16

**Authors:** Mototsugu Oya, Shuichi Kaneko, Tsuneo Imai, Toshiaki Tsujino, Toshiyuki Sunaya, Yutaka Okayama

**Affiliations:** 1 Department of Urology, Keio University School of Medicine, Tokyo, Japan; 2 Department of Gastroenterology, Kanazawa University Hospital, Kanazawa, Japan; 3 National Hospital Organization, Higashi Nagoya National Hospital, Nagoya, Japan; 4 Medical Affairs and Pharmacovigilance, Bayer Yakuhin, Ltd., 2-4-9 Umeda, Kita-Ku, Osaka, 530-0001 Japan; 5 Product Development, Bayer Yakuhin, Ltd., Osaka, Japan

**Retraction Note: Cancer Chemotherapy and Pharmacology (2022) 89:761-772** 10.1007/s00280-022-04428-0

The Authors have retracted this article. After publication the authors found a flaw in the way the raw data was handled for this study and found that the data analysis was incomplete. The authors re-analysed the data and found that the conclusions initially drawn from the results were no longer supported. The authors, therefore, no longer have confidence in the results presented in this article. All authors agree to this retraction.

